# Identification of Emerging Human Mastitis Pathogens by MALDI-TOF and Assessment of Their Antibiotic Resistance Patterns

**DOI:** 10.3389/fmicb.2017.01258

**Published:** 2017-07-12

**Authors:** María Marín, Rebeca Arroyo, Irene Espinosa-Martos, Leónides Fernández, Juan M. Rodríguez

**Affiliations:** ^1^Departamento de Nutrición, Bromatología y Tecnología de los Alimentos, Universidad Complutense de Madrid Madrid, Spain; ^2^Probisearch Madrid, Spain

**Keywords:** human mastitis, breastfeeding, mastitis pathogens, antibiotic resistance, MALDI-TOF, antimicrobial susceptibility testing

## Abstract

Lactational mastitis constitutes one of the main causes of undesired weaning, depriving the mother–infant pair from the benefits of breastfeeding; therefore, this condition should be considered a relevant public health issue. The role of specific microorganisms remains unclear since human milk cultures and antibiotic susceptibility testing (AST) are not routinely performed, despite the fact that this would be key to ensure an early and effective diagnosis and treatment. The objective of this study was to describe the culturable microbial diversity in 647 milk samples from breastfeeding women with clinical symptoms of mastitis by Matrix-Assisted Laser Desorption Ionization Time-of-Flight Mass Spectrometry (MALDI-TOF) VITEK MS technology and to analyze the antimicrobial susceptibility profiles of a collection of isolates from these samples by the VITEK 2 AST system. *Staphylococcus epidermidis* was the most common species isolated from mastitis samples (87.6%), while *Staphylococcus aureus* was detected in 22.1%. Streptococci constituted the second (68.6%) most prevalent bacterial group, with *Streptococcus mitis/oralis, Streptococcus salivarius*, and *Streptococcus parasanguinis* detected with frequencies of 40.8, 36.8, and 14.4%, respectively. The antibiotic susceptibility profiles of 642 staphylococcal isolates indicated a remarkable resistance to benzylpenicillin (88.3%) and erythromycin (67.3%) with differences between species. A high percentage of *Staphylococcus* isolates were resistant to at least one antibiotic (*Staphylococcus hominis*, 100%; *S. epidermidis*, 98.2%; *S. aureus*, 92.9%; *Staphylococcus lugdunensis*, 90.5%) and the percentage of multidrug-resistance (MDR) isolates was noticeable (*S. hominis*, 81%; *S. epidermidis*, 64.4%; *S. aureus*, 11.5%; *S. lugdunensis*, 10.5%). In relation to streptococcal isolates (*n* = 524), AST revealed high or moderate percentages of resistance to erythromycin (68.7%), benzylpenicillin (63.7%), ampicillin (51.5%), and tetracycline (30.8%). Antibiotic resistance to at least one antibiotic was detected in 97.6% of *S. parasanguinis*, 92.6% of *S. salivarius*, 83.3% of *S. mitis/oralis*, and 72.4% of *Streptococcus vestibularis* isolates. A significant number of MDR streptococcal isolates was also found (*S. parasanguinis*, 51.2%; *S. salivarius*, 39.3%; *S. mitis/oralis*, 34.6%; and *S. vestibularis*, 19%). The results highlight the important role of coagulase-negative staphylococci and streptococci as human mastitis-causing agents. Moreover, the high rates of antimicrobial resistance among these microorganisms must be contemplated as an issue of clinical relevance in relation to treatment options.

## Introduction

Infectious mastitis is a common condition that affects up to 33% of women during lactation and constitutes one of the main causes of undesired weaning (WHO, 2000; Foxman et al., 2002; Scott et al., 2008; Amir and Academy of Breastfeeding Medicine Protocol Committee, 2014), depriving the mother–infant pair from the health benefits of breastfeeding (U.S. Department of Health and Human Services, 2011). This pathological condition is characterized by a dysbiosis process in the mammary gland, leading to an overgrowth of certain bacterial species present in human milk (Delgado et al., 2008; Contreras and Rodríguez, 2011; Fernández et al., 2013). However, the role of specific microorganisms still remains unclear since human milk cultures and antibiotic susceptibility testing (AST) are not routinely performed (Scott et al., 2008; Barlow, 2011; Contreras and Rodríguez, 2011). The lack of this information prompts the empiric use of broad-spectrum antibiotics giving rise to a frequent therapeutic failure (Fernández and Rodríguez, 2014). In this sense, a rapid and reliable identification of the microorganisms involved in mastitis together with the determination of their antimicrobial susceptibility profile is relevant to ensure an early and effective diagnosis and treatment, leading to a decrease in antimicrobial resistance rates and lower treatment costs.

Until recently, microbial identification in clinical microbiology laboratories has mostly relied on conventional methods that involve culturing, morphologic phenotyping, and biochemical testing. These procedures often require large amounts of biological materials, are time-consuming and the species assignment may be imprecise. Molecular identification methods mainly based on the sequencing of housekeeping genes, such as 16S rRNA, are more discriminating than phenotypic tests, but also more complicated and expensive; therefore, they are not ideal for application on a routine basis in clinical laboratories. In the last years, the application of matrix-assisted laser desorption ionization-time of flight mass spectrometry (MALDI-TOF MS) technology has emerged as an accurate, rapid and cost-effective tool for the identification of clinically relevant microorganisms in large laboratories, providing a valuable alternative to phenotypic and molecular methods (Bizzini et al., 2011; Nomura, 2015; Singhal et al., 2015). This approach has been used successfully to identify bovine mastitis pathogens (Tomazi et al., 2014; Schmidt et al., 2015); however, it has been scarcely applied to date for the identification of microorganisms causing human mastitis.

Different commercial systems have been developed for AST due to their ease of use and interpretation, speed and cost-effectiveness in relation to the laborious standard methods of broth microdilution and disk diffusion. Among them, VITEK 2 (bioMérieux, Marcy l’Étoile, France) is an automated AST system that provides determinations of the minimum inhibitory concentration (MIC) within a few hours after isolation and integrates an expert system (Advanced Expert System) for biological validation of susceptibility results and therapeutic interpretation (Winstanley and Courvalin, 2011).

In this context, the objective of this study was to describe the microbial diversity in 647 milk samples from breastfeeding women with clinical symptoms of mastitis by MALDI-TOF VITEK MS technology and to analyze the antimicrobial susceptibility profiles of a collection of 1,180 mastitis isolates obtained from these samples by the VITEK 2 AST system.

## Materials and Methods

### Isolation of Bacteria from Milk Samples

Milk samples (*n* = 647) were obtained from women with clinical symptoms of infectious mastitis. All cases included either both local (breast redness, pain, engorgement, and reduced milk secretion) and systemic symptoms (fever or flu-like symptoms) or only local symptoms. Written informed consent to the protocol (reference 10/017E) approved by the Ethical Committee of Hospital Clínico San Carlos (Madrid, Spain) was obtained from the women that provided the biological samples of this study. In the case of women with previous antibiotic therapy (33%, based on patient self-report of antibiotic use), samples were collected at least 2 weeks after finishing the treatment. Milk samples for microbial analysis were collected following the protocol proposed by Delgado et al. (2015). Briefly, nipple and mammary areola were cleaned with soap and water and a milk sample (∼1 ml) was collected aseptically in a sterile tube by manual expression after discarding the first drops. The use of milk pumps for sample collection was absolutely discouraged to avoid contamination. Milk samples were plated onto ready-to-use Columbia nalidixic acid agar plates (bioMérieux, Madrid, Spain) and incubated at 37°C for 48 h. In order to reach a positive diagnosis of mastitis, the presence of different bacterial morphotypes was investigated and the following microbiological criteria were established: coagulase-negative staphylococci, viridans group streptococci (VGS), and other species > 1,000 CFU/ml; *Staphylococcus aureus* and *Corynebacterium* spp. > 150 CFU/ml, since the latter could lead to mastitis at much lower concentrations.

### Identification by MALDI-TOF Mass Spectrometry

A total of 2,323 isolates from the mastitis milk samples were identified by MALDI-TOF using a VITEK MS instrument (bioMérieux, Marcy l’Etoile, France) in the facilities of Probisearch (Tres Cantos, Madrid, Spain). Briefly, a portion of a bacterial colony (∼1 μL) was spotted onto a MALDI sample plate, overlaid with 1 μL of a saturated solution of α-cyano-4-hydroxycinnamic acid in acetonitrile (28%), and then allowed to dry at room temperature. For each isolate, a mean spectrum was constructed with at least 50 *m/z* spectra profiles and used for the identification by comparison with the spectra contained in the Myla database (bioMérieux). Identification was defined as a 99–100% match to the species-specific *m*/*z* profiles in the database.

### Antimicrobial Susceptibility Testing

A total of 1,180 staphylococcal, streptococcal and enterococcal isolates, previously identified at species level by MALDI-TOF, were subjected to AST with the VITEK 2 technology (bioMérieux). The *Staphylococcus* isolates (*n =* 642) belonged to *Staphylococcus aureus* (*n* = 140), *Staphylococcus epidermidis* (*n* = 435), *Staphylococcus hominis* (*n* = 21), *Staphylococcus lugdunensis* (*n* = 21), and other minority species (*n* = 25). The *Streptococcus* isolates (*n =* 524) belonged to *Streptococcus mitis/oralis* (*n* = 215), *Streptococcus parasanguinis* (*n* = 82), *Streptococcus salivarius* (*n* = 162), *Streptococcus vestibularis* (*n* = 29) and other minority species (*n* = 36). MALDI-TOF MS Vitek instrument was unable to discriminate between the species *S. mitis* and *S. oralis*. All enterococal isolates (*n =* 14) belonged to *Enterococcus faecalis*. The isolates were subcultured on Columbia nalidixic acid agar plates and incubated at 37°C for 48 h. Colonies were resuspended in 0.45% NaCl solution and the bacterial suspension was adjusted to the turbidity of 0.5–0.6 McFarland standard with an ATB 1550 densitometer (bioMérieux) and properly diluted to inoculate the test cards for VITEK 2 according to the manufacturer’s instructions. The following test cards (bioMérieux) were used: AST-P626 (*Staphylococcus* spp.), AST-ST01 (VGS and *Streptococcus pyogenes*) and AST-P589 (*Enterococcus* spp. and *Streptococcus agalactiae*). Antimicrobial concentration ranges and quality control strains of these cards were established according to the Clinical and Laboratory Standards Institute (CLSI) recommendations (Clinical and Laboratory Standards Institute [CLSI], 2013). The resulting MIC values were categorized into clinical categories of susceptible, intermediate or resistant following the interpretive criteria of the VITEK 2 expert system based on the CLSI guidelines (Clinical and Laboratory Standards Institute [CLSI], 2013). Isolates with MIC in the intermediate range were classified as resistant for data analysis. Resistance to, at least, one antimicrobial and multidrug-resistance (MDR) were calculated for the main *Staphylococcus* and *Streptococcus* species isolated from mastitic milk. MDR was defined as acquired non-susceptibility to at least one agent in three or more antimicrobial categories. In the case of *S. aureus*, oxacillin or cefoxitin-resistant isolates were considered always as harboring MDR (Magiorakos et al., 2012).

### Statistical Analysis

Microbiological data, recorded as CFU/ml, were transformed to logarithmic values before statistical analysis. The reported values of bacterial counts are the mean and 95% CI values of the mean. Proportions to determine associations between bacterial species were compared using χ^2^ statistics. The frequencies of the susceptibility data for each antibiotic and microorganisms species were analyzed using a χ^2^ test followed by the Bonferroni *post hoc* pair-wise comparison. All analyses were conducted with a significance level of *p* < 0.05. Collected data were analyzed using the statistical software StatGraphics Centurion XVI version 16.2.04 (StatPoint Technologies, Inc., Warrenton, VA, United States) and R 2.15.3 (R-project^1^).

## Results

### Microbiological Analysis of Milk Samples and Identification of Isolates by MALDI-TOF MS

In this study, a total of 647 milk samples from women suffering infectious mastitis were analyzed using culture-based methods. Bacterial growth was observed in all analyzed samples with a mean value of 4.00 log_10_ CFU/ml (95% CI 3.94–4.06) and ranging from 1.70 to 6.21 log_10_ CFU/ml. A total of 2,323 isolates were identified by MALDI-TOF VITEK MS, 95.1% at the species level and the remaining 4.9% at the genus level. The results are summarized in **Table 1**.

**Table 1:** Microbiological analysis of milk samples from women suffering mastitis (*n* = 647).

Microorganism	No. samples^a^	Frequency (%)	Mean (95% CI) (log_10_ CFU/mL)	Range [min, max] (log_10_ CFU/mL)
*Staphylococcus epidermidis*	567	87.6	3.56 (3.49–3.62)	4.48 [1.70, 6.18]
*Staphylococcus aureus*	143	22.1	3.65 (3.47–3.83)	4.40 [1.70, 6.10]
*Staphylococcus hominis*	34	5.3	3.46 (3.12–3.80)	3.98 [1.70, 5.68]
*Staphylococcus lugdunensis*	21	3.3	3.18 (2.75–3.58)	3.30 [1.70, 5.00]
*Staphylococcus warneri*	11	1.7	2.79 (2.61–2.97)	0.88 [2.30, 3.18]
*Staphylococcus pasteuri*	10	1.6	3.28 (2.79–3.78)	2.12 [2.18, 4.30]
Other staphylococci^b^	16	2.5	3.02 (2.64–3.42)	2.60 [1.70, 4.30]
Total staphylococci	609	94.1	3.70 (3.63–3.77)	4.51 [1.70, 6.21]
*Rothia mucilaginosa*	112	17.3	2.62 (2.49–2.75)	2.78 [1.70, 4.48]
*Rothia* spp.	35	5.4	2.52 (2.24–2.79)	3.00 [1.70, 4.70]
Total *Rothia*	144	22.3	2.61 (2.49–2.72)	3.00 [1.70, 4.70]
*Streptococcus mitis/oralis*	264	40.8	3.27 (3.17–3.37)	4.30 [1.70, 6.00]
*Streptococcus parasanguinis*	93	14.4	3.29 (3.14–3.44)	3.30 [1.70, 5.00]
Mitis group streptococci	318	49.2	3.35 (3.26–3.44)	4.30 [1.70, 6.00]
*Streptococcus salivarius*	238	36.8	3.05 (2.95–3.14)	3.54 [1.70, 5.24]
*Streptococcus vestibularis*	32	5.0	3.48 (3.12–3.83)	4.34 [1.70, 6.04]
Salivarius group streptococci	255	39.4	3.12 (3.02–3.21)	4.34 [1.70, 6.04]
Other streptococci^c^	68	10.5	3.36 (3.14–3.59)	4.70 [1.70, 6.40]
Total streptococci	444	68.6	3.47 (3.40–3.55)	4.70 [1.70, 6.40]
*Corynebacterium tuberculostearicum*	23	3.6	2.30 (1.97–2.63)	2.45 [1.70, 4.15]
*Corynebacterium kroppenstedtii*	13	2.0	2.17 (1.94–2.41)	1.00 [1.70, 2.70]
Other corynebacteria^d^	46	7.1	2.95 (2.65–3.25)	3.28 [1.70, 4.98]
Total corynebacteria	75	11.6	2.65 (2.44–2.87)	3.27 [1.70, 4.98]
Enterococci^e^	24	3.7	3.16 (2.66–3.67)	3.48 [1.70, 5.18]
Total microorganisms	647	100	4.00 (3.94–4.06)	4.52 [1.70, 6.21]

*^a^Number of samples with presence of the species. A total of 2,323 isolates were identified by MALDI-TOF MS. ^b^Other staphylococci (16 isolates): S. capitis (7), S. haemolyticus (5), S. pseudintermedius (1), Staphylococcus spp. (3). ^c^Other streptococci (68 isolates): S. agalactiae (9), S. anginosus (5), S. dysgalactiae (2), S. gallolyticus (1), S. mutans (1), S. pyogenes (5), Streptococcus spp. (45). ^d^Other corynebacteria (46 isolates): C. mucifaciens (9), C. aurimucosum (3), C. pseudodiphtheriticum (3), C. jeikenium (2), C. xerosis/amycolatum (2), Corynebacterium spp. (28). ^e^Enterococci (24 isolates): Enterococcus faecalis (16), E. faecium (4), Enterococcus spp. (4).*

*Staphylococcus* was the most frequently isolated bacterial genus, since a total of 609 milk samples (94.1%) contained at least one isolate of this genus. The total staphylococcal counts found in the milk samples presented a mean value of 3.70 log_10_ CFU/ml (95% CI 3.63–3.77). *S. epidermidis* was the most common species isolated from mastitis samples in this study (87.6%), while *S. aureus, S. hominis*, and *S. lugdunensis* were isolated in 22.1, 5.3, and 3.3% of them, respectively. The mean bacterial counts for *S. epidermidis* and *S. aureus* were 3.56 (95% CI 3.49–3.62) and 3.65 log_10_ CFU/ml (95% CI 3.47–3.83), respectively. *Rothia* was present in 22.3% of the samples, being *Rothia mucilaginosa* the species most commonly isolated of this genus with a detection frequency of 17.3%.

The total streptococcal counts in the samples had a mean value of 3.47 log_10_ CFU/ml (95% CI 3.40–3.55) and oscillated between 1.70 and 6.40 log_10_ CFU/ml. This genus was isolated from 68.6% of the samples and, therefore, constitutes the second most prevalent bacterial group among the samples of mastitis milk analyzed in this study. Streptococcal isolates belonging to the mitis group were detected in 49.2% of the samples, with *S. mitis/oralis* (40.8%) and *S. parasanguinis* (14.4%) as the most common species of this group. Salivarius group streptococci were present in 39.4% of the samples, and the species *S. salivarius* and *S. vestibularis* had a detection frequency of 36.8 and 5%, respectively.

Species belonging to the genus *Corynebacterium* were found in 11.6% of mastitis specimens, with a mean value of 2.65 log_10_ CFU/ml (95% CI 2.44–2.87). *Corynebacterium tuberculostearicum* (3.6%) and *Corynebacterium kroppenstedtii* (2%) were the corynebacterial species most frequently isolated in this study. Finally, enteroccocci were isolated in 3.7% of samples, being *E. faecalis* the most common species of this group.

### Antimicrobial Susceptibility Testing

The antibiotic susceptibility profiles of 642 staphylococcal isolates are presented in **Table 2**. The MIC of the tested antimicrobial agents against *S. aureus* and *S. epidermidis* are compiled in Supplementary Tables S1, S2 and Figures S1, S2. Globally, the staphylococcal isolates displayed a remarkable resistance to benzylpenicillin (88.3%) and erythromycin (67.3%), although there were some differences depending on the species (**Table 2**). *S. aureus* isolates showed susceptibility (90–100%) to most of the other antimicrobials tested, while *S. epidermidis* ones exhibited a noticeable percentage of resistance against oxacillin (46%), cefoxitin (46%), fusidic acid (41.4%), and mupirocin (28.7%). No strains resistant to linezolid or tigecycline were found and the susceptibility to the rest of antibiotics tested was between 78.6 and 99.8%. A significant difference between S. *aureus* and *S. epidermidis* isolates was detected for resistance to oxacillin, fusidic acid, mupirocin, and cefoxitin (χ^2^ test, *p* < 0.001), and benzylpenicillin, gentamycin, and clindamycin (χ^2^ test, *p* < 0.05). In addition to benzylpenicillin (85.7%) and erythromycin (85.7%), *S. hominis* isolates demonstrated remarkable resistance to fosfomycin (81%), and moderate resistance to fusidic acid (38.1%), oxacillin (33.3%), cefoxitin (28.6%), and clindamycin (23.8%). Most of this species isolates were susceptible to the rest of the antimicrobials. All the isolates belonging to *S. lugdunensis* displayed high susceptibility against all the antibiotics tested (81–100%), excepting benzylpenicillin (81% resistant isolates).

**Table 2:** Antibiotic susceptibility profiles of *S. aureus, S. epidermidis*, and other *Staphylococcus* species isolated from milk samples from women suffering infectious mastitis.

Antibiotic		All isolates (*n* = 642)	*S. aureus* (*n* = 140)	*S. epidermidis* (*n* = 435)	*S. hominis* (*n* = 21)	*S. lugdunensis* (*n* = 21)	Other^∗^ (*n* = 25)
Benzylpenicillin	R	88.3	81.4^†^	92.9^†^	85.7	81	54.2
	S	11.7	18.6	7.1	14.3	19	45.8
Oxacillin	R	32.8	2.9^‡^	46^‡^	33.3	0	0
	S	67.2	97.1	54	66.7	100	100
Gentamycin	R	3.9	0^†^	5.8^†^	0	0	0
	S	96.1	100	94.2	100	100	100
Tobramycin	R	5.3	1.4	7.4	0	0	0
	S	94.7	98.6	92.6	100	100	100
Levofloxacin	R	4.4	1.4	6	0	0	0
	S	95.6	98.6	94	100	100	100
Erythromycin	R	67.3	65.7	71.5	85.7	19	28
	S	32.7	34.3	28.5	14.3	81	72
Clindamycin	R	17.9	10^†^	21.4^†^	23.8	14.3	0
	S	82.1	90	78.6	76.2	85.7	100
Linezolid	R	0	0	0	0	0	0
	S	100	100	100	100	100	100
Daptomycin	R	0.2	0	0.2	0	0	0
	S	99.8	100	99.8	100	100	100
Teicoplanin	R	1.6	0	2.3	0	0	0
	S	98.4	100	97.7	100	100	100
Vancomycin	R	0.3	0	0.5	0	0	0
	S	99.7	100	99.5	100	100	100
Tigecycline	R	0.2	0	0	4.8	0	0
	S	99.8	100	100	95.2	100	100
Fosfomycin	R	12.1	2.9	6.9	81	14.3	96
	S	87.9	97.1	93.1	19	85.7	4
Fusidic acid	R	29.9	0^‡^	41.4^‡^	38.1	4.8	12
	S	70.1	100	58.6	61.9	95.2	88
Mupirocin	R	20.2	0^‡^	28.7^‡^	19	0	4
	S	79.8	100	71.3	81	100	96
Rifampicin	R	0.2	0	0.2	0	0	0
	S	99.8	100	99.8	100	100	100
TMP-SMZ	R	2.2	0	2.8	9.5	0	0
	S	97.8	100	97.2	90.5	100	100
Cefoxitin	POS	32.7	2.9^‡^	46^‡^	28.6	0	0
	NEG	67.3	97.1	54	71.4	100	100
ICR	POS	6.9	8.6	6.7	9.5	4.8	0
	NEG	93.1	91.4	93.3	90.5	95.2	100

*R, resistant isolates (%); S, susceptible isolates (%). Isolates were categorized as susceptible or resistant by *Clinical and Laboratory Standards Institute* criteria (Clinical and Laboratory Standards Institute [CLSI], 2013). Isolates in the intermediate range were classified as resistant for data analysis. TMP-SMZ, trimethoprim-sulfamethoxazole; ICR, inducible clindamycin resistance; POS, positive; NEG, negative. ^∗^Other staphylococci (25 isolates): S. capitis (6), S. pasteuri (7), S. haemolyticus (5), S. warneri (6), S. pseudintermedius (1). Chi-square (χ^*2*^) test was used to compare the difference in the antibiotic susceptibility pattern between S. aureus and S. epidermidis. ^†^*p* < 0.05; ^‡^*p* < 0.001.*

**FIGURE 1:**
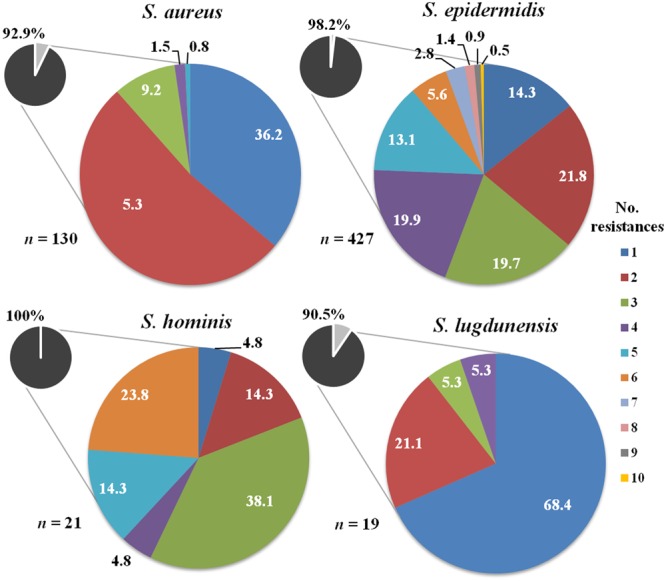
Number of resistances to the antibiotics tested among *Staphylococcus* isolates recovered from mastitis milk samples. The total percentage of isolates resistant to one or more antibiotics in each species is shown in small circles.

The percentage of *Staphylococcus* isolates resistant to at least one antimicrobial and the number of resistances exhibited by these isolates was calculated for the most important species (**Figure 1**). The results indicated that a remarkable percentage of *Staphylococcus* isolates were resistant to at least one antibiotic: *S. aureus* (92.9%), *S. epidermidis* (98.2%), *S. hominis* (100%), and *S. lugdunensis* (90.5%). *S. epidermidis* presented the highest number of antibiotic resistances (between 1 and 10), followed by *S. hominis* (1-6), *S. aureus* (1-5), and *S. lugdunensis* (1-4). The percentage of MDR isolates was noticeable in the case of *S. hominis* (81%) and *S. epidermidis* (64.4%). In relation to *S. aureus* and *S. lugdunensis*, 11.5 and 10.5% of MDR isolates were found, respectively.

Antimicrobial susceptibility tests were performed on a total of 524 streptococcal isolates and the results are summarized in **Table 3**. The MIC of the tested antimicrobial agents against *S. mitis/oralis, S. parasanguinis*, and *S. salivarius* are also presented in Supplementary Tables S3–S5 and Figures S3–S5. Overall, the streptococcal isolates showed high or moderate percentages of resistance to erythromycin (68.7%), benzylpenicillin (63.7%), ampicillin (51.5%) and tetracycline (30.8%), and susceptibility rates between 83.9 and 99.8% to the rest of the antimicrobials tested. *S. mitis/oralis* demonstrated a noteworthy resistance against erythromycin (69.6%) and benzylpenicillin (56.7%), followed by ampicillin (42.8%) and tetracycline (30.7%). Most of the isolates were sensitive to cefotaxime (82.8%), ceftriaxone (84.2%), clindamycin (89.3%), levofloxacin (96.3%), and linezolid (99.5%). No vancomycin-resistant strains were identified. In comparison to *S. mitis/oralis* isolates, *S. parasanguinis* ones exhibited a higher percentage of resistance to erythromycin (80.8%), benzylpenicillin (80.5%), ampicillin (72%), and tetracycline (51.2%) and a slightly lower susceptibility to cefotaxime (79.3%), ceftriaxone (81.7%), clindamycin (82.9%), and levofloxacin (85.4%). No strains resistant to linezolid or vancomycin were found. *S. salivarius* also displayed remarkable resistance to benzylpenicillin (76.4%), erythromycin (70.4%), ampicillin (60.9%), and a noticeable sensitivity (77.8–100%) to the rest of antibiotics assayed. Regarding *S. vestibularis*, 41.4% of isolates were erythromycin-resistant; however, the susceptibility to the rest of antimicrobials tested was high (72.4–100%). In fact, no strains resistant to cefotaxime, ceftriaxone, or linezolid appeared among the isolates of this species.

**Table 3:** Antibiotic susceptibility profiles of *Streptococcus* species isolated from milk samples from women suffering infectious mastitis.

Antibiotic		All isolates (*n* = 524)	*S. mitis/oralis* (*n* = 215)^‡^	*S. parasanguinis* (*n* = 82)^‡^	*S. salivarius* (*n* = 162)^‡^	*S. vestibularis* (*n* = 29)	Other^∗^ (*n* = 36)
Benzylpenicillin	R	63.7	56.7^a^	80.5^b^	76.4^b^	27.6	38.9
	S	36.3	43.3	19.5	23.6	72.4	61.1
Ampicillin	R	51.5	42.8^a^	71.9^b^	60.9^b^	24.1	37.1
	S	48.5	57.2	28	39.1	75.9	62.9
Cefotaxime	R	14.8	17.2	20.7	9.9	0	24
	S	85.2	82.8	79.3	90.1	100	76
Ceftriaxone	R	13	15.8	18.3	8	0	19.2
	S	87	84.2	81.7	92	100	80.8
Levofloxacin	R	12	3.7^a^	14.6^b^	22.2^c^	20.7	2.8
	S	88	96.3	85.4	77.8	79.3	97.2
Erythromycin	R	68.7	69.6	80.8	70.4	41.4	48.3
	S	31.3	30.4	19.2	29.6	58.6	51.7
Clindamycin	R	16.1	10.7	17.1	21.6	13.8	36.1
	S	83.9	89.3	82.9	78.4	86.2	63.9
Linezolid	R	0.2	0.5	0	0	0	0
	S	99.8	99.5	100	100	100	100
Vancomycin	R	1	0	0	2.5	3.4	0
	S	99	100	100	97.5	96.6	100
Tetracycline	R	30.8	30.7^a^	51.2^b^	21^a^	17.2	38.5
	S	69.2	69.3	48.8	79	82.8	61.5

*R, resistant isolates (%); S, susceptible isolates (%). Isolates were categorized as susceptible or resistant by *Clinical and Laboratory Standards Institute* criteria (Clinical and Laboratory Standards Institute [CLSI], 2013). Isolates in the intermediate range were classified as resistant for data analysis. ^∗^Other streptococci (36 isolates): S. agalactiae (9), S. anginosus (5), S. cristatus (2), S. dysgalactiae (1), S. gallolyticus (1), S. gordonii (5), S. pyogenes (5), S. pneumoniae (3), S. sanguinis (5). Data for S. agalactiae resistance against cefotaxime, ceftriaxone, and tetracyline are not included since these antimicrobials are not tested in the AST-P589 card. ^‡^Different lowercase superscripts denote a significant difference (χ^*2*^ test; *p* < 0.001) in the antibiotic susceptibility pattern between S. mitis/oralis, S. parasanguinis, and S. salivarius.*

**FIGURE 2:**
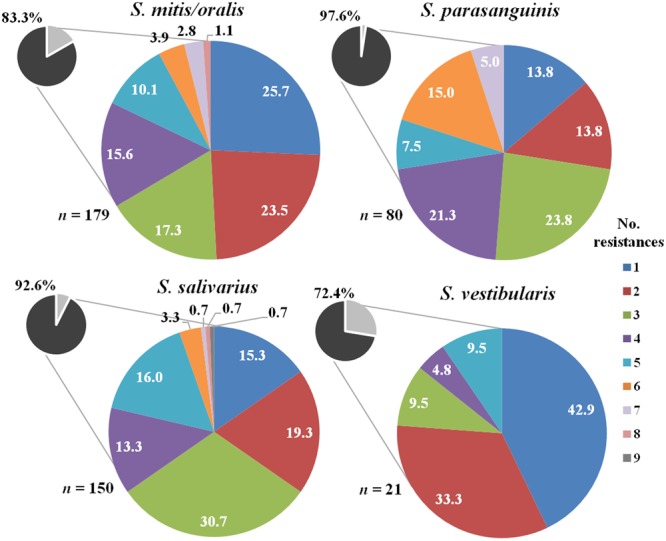
Number of resistances to the antibiotics tested among *Streptococcus* isolates recovered from mastitis milk samples. The total percentage of isolates resistant to one or more antibiotics in each species is shown in small circles.

As shown in **Figure 2**, a high percentage of *Streptococcus* isolates from human mastitis samples were also resistant to at least one antibiotic: *S. mitis/oralis* (83.3%), *S. parasanguinis* (97.6%), *S. salivarius* (92.6%), and *S. vestibularis* (72.4%). *S. salivarius* presented the highest number of antibiotic resistances (between 1 and 9), followed by *S. mitis/oralis* (1-8), *S. parasanguinis* (1-7), and *S. vestibularis* (1-5). The percentage of MDR isolates was significant in the case of *S. parasanguinis* (51.2%), *S. salivarius* (39.3%) and *S. mitis/oralis* (34.6%). Approximately, 19% of *S. vestibularis* isolates exhibited MDR.

**FIGURE 3:**
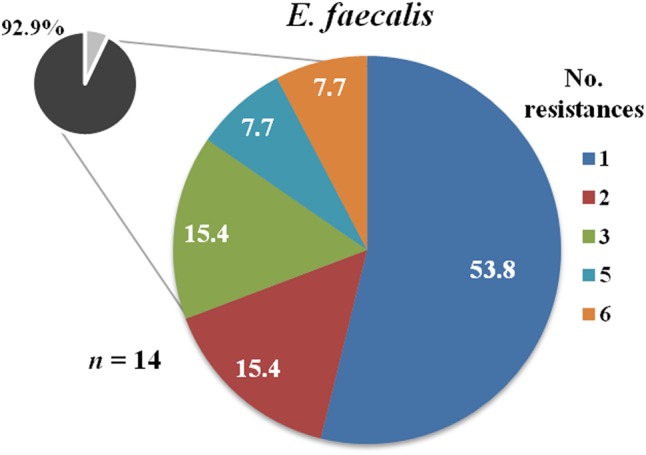
Number of resistances to the antibiotics tested among *Enterococcus faecalis* isolates recovered from mastitis milk samples. The total percentage of isolates resistant to one or more antibiotics is shown in the small circle (intrinsic resistances to cefuroxime, cefuroxime-axetil, clindamycin, trimethoprim-sulfamethoxazole, and quinupristin-dalfopristin are excluded).

Regarding the genus *Enterococcus, E. faecalis* isolates (*n* = 14) were not sensitive to those antibiotics to which they are intrinsically resistant (cefuroxime, cefuroxime-axetil, clindamycin, trimethoprim-sulfamethoxazole, and quinupristin-dalfopristin) (**Table 4**) and 85.7% of them were resistant to erythromycin. Approximately, 20% of the *E. faecalis* isolates displayed resistance to high levels of aminoglycosides (gentamycin, kanamycin, streptomycin) and around 7% was resistant to quinolones (ciprofloxacin and levofloxacin) and glycopeptides (vancomycin and teicoplanin). Globally, 93% of *E. faecalis* isolates were resistant to, at least, one antibiotic (excluding intrinsic resistance) and 21.4% were MDR (**Figure 3**).

**Table 4:** Antibiotic susceptibility profiles of *Enterococcus faecalis* isolated from human mastitis.

Antibiotic		*E. faecalis* (*n* = 14)
Benzylpenicillin	R	7.1
	S	92.9
Ampicillin	R	7.7
	S	92.3
Cefuroxime	R	100
	S	0
Cefuroxime-axetil	R	100
	S	0
Imipenem	R	0
	S	100
Gentamycin HL	R	16.7
	S	83.3
Kanamycin HL	R	23.1
	S	76.9
Streptomycin HL	R	25
	S	75
Ciprofloxacin	R	7.7
	S	92.3
Levofloxacin	R	7.1
	S	92.9
Erythromycin	R	85.7
	S	14.3
Clindamycin	R	100
	S	0
QD	R	100
	S	0
Linezolid	R	0
	S	100
Teicoplanin	R	7.1
	S	92.9
Vancomycin	R	7.7
	S	92.3
Tigecycline	R	0
	S	100
Nitrofurantoin	R	7.7
	S	92.3
Chloramphenicol	R	0
	S	100
TMP-SMZ	R	100
	S	0

*R, resistant isolates (%); S, susceptible isolates (%); HL, high level; QD, quinupristin-dalfopristin; TMP-SMZ, trimethoprim-sulfamethoxazole.*

## Discussion

Mastitis is a common reason for antibiotic treatment in lactating women. In fact, studies carried out in industrialized countries have reported high rates of antibiotic prescriptions for mastitis (Foxman et al., 2002; Scott et al., 2008), despite the fact that this empiric practice may be a relevant risk factor for acute or subacute episodes to become chronic or recurrent due to the selection of antibiotic resistant strains (Mediano et al., 2014; Fernández et al., 2016). In the absence of microbiological analysis and antimicrobial susceptibility testing, the antibiotic treatment usually depends on physician choice rather than on scientific evidence, leading to the increase of antimicrobial resistance among mastitis-causing agents (Barlow, 2011; Jahanfar et al., 2013).

It is widely accepted that antibiotics are responsible for dysbiosis processes in human microbiota leading to antibiotic-associated diarrhea and gastroenteritis, urogenital and oral infections. Host–microbiota interactions are dynamic and, therefore, changes in the microbiota as a consequence of antibiotic treatment can result in the dysregulation of host immune homeostasis and an increased susceptibility to infectious, allergic and inflammatory diseases (Joffe and Simpson, 2009; Willing et al., 2011). In the last few years, it is becoming evident that antibiotic therapy during pregnancy, intrapartum or lactation affects the maternal (intestinal, vaginal, mammary) microbiota (Soto et al., 2014; Gonçalves et al., 2016), which alters normal mother-to-infant transmission of microorganisms, affecting the development of the infant intestinal microbiota and having negative consequences to infant health (Murk et al., 2011; Stensballe et al., 2013; Gibson et al., 2015; Rutten et al., 2015; Mazzola et al., 2016).

Nowadays, the most commonly prescribed antibiotics for mastitis are penicillins, cephalosporins, erythromycin, and clindamycin (Foxman et al., 2002; Eglash et al., 2006; Scott et al., 2008; Barlow, 2011; Amir and Academy of Breastfeeding Medicine Protocol Committee, 2014; Witt et al., 2014); however, there is limited consensus on which patients should receive antibiotics, which one is the most appropriate, when to initiate therapy, its duration or the dosage and posology (Jahanfar et al., 2013; Amir and Academy of Breastfeeding Medicine Protocol Committee, 2014). Moreover, the impact of antibiotic therapy on the host microbiota and on the development of antimicrobial resistance among potential human mastitis pathogens has been scarcely evaluated to date.

Staphylococci are the most common pathogens causing human mastitis (Lawrence and Lawrence, 2011); *S. aureus* is the main etiological agent of acute mastitis and breast abscesses (Reddy et al., 2007; Delgado et al., 2011; Branch-Elliman et al., 2013), while coagulase-negative staphylococci are usually related to subacute or subclinical infection. Among the latter, *S. epidermidis* is emerging as the leading cause of mastitis worldwide (Contreras and Rodríguez, 2011). In this sense, the results of the present study confirm the important role that *S. epidermidis* plays as an etiologic agent in human mastitis, in agreement with previous studies (Delgado et al., 2008, 2009; Mediano et al., 2017).

There are limited studies reporting the antimicrobial susceptibility of staphylococci isolates recovered from milk of women suffering mastitis. In the present study, antimicrobial resistance among the *S. aureus* isolates was less common than among the *S. epidermidis* ones, although the resistance to benzylpenicillin and erythromycin among all staphylococcal isolates was remarkable. First generation penicillins, such as amoxicillin, and the macrolide erythromycin have been recommended for years (WHO, 2000), but nowadays their use is discouraged due to the increasing resistance to these antibiotics among *S. aureus* isolates (Scott et al., 2008). Clinicians should also be aware of the rise of methicillin-resistant *Staphylococcus aureus* (MRSA) isolated in cases of mastitis and breast abscesses (Reddy et al., 2007; Branch-Elliman et al., 2013). However, in this study, *S. aureus* isolates showed high susceptibility to oxacillin, in accordance with a previous work (Delgado et al., 2011). Furthermore, among the isolates of *S. aureus* that exhibited some resistance, about 12% were MDR, in contrast to *S. epidermidis* (64%). The evolution of antibiotic resistance among *S. aureus* involved in mastitis should be followed up since this species has a remarkable ability to acquire multi-antibiotic resistance (Hiramatsu et al., 2014).

Penicillin resistance is the most common antimicrobial resistance in pathogenic *S. epidermidis* (Bansal et al., 2015; Taponen et al., 2016). In this study, the highest antibiotic resistance of this species was observed against benzylpenicillin, which is conferred by the production of β-lactamases, but the resistance to oxacillin, which is resistant to these enzymes, must be highlighted. In this sense, rates of methicillin resistance in *S. epidermidis* have increased steadily during the last decades (Becker et al., 2014), which is of special concern because *mec* genes may be transferred between staphylococcal species and constitute a risk for public health (Wendlandt et al., 2013; Taponen et al., 2016). Delgado et al. (2009) characterized the antimicrobial susceptibility profiles of 40 *S. epidermidis* strains from human mastitis and showed a high percentage of resistance to oxacillin, erythromycin, clindamycin and mupirocin, in agreement with the results of the present study. Similar resistance frequency for oxacillin and clindamycin have been described in the case of coagulase-negative staphylococci isolated from milk of lactating women with chronic breast pain receiving oral antibiotic therapy (Witt et al., 2014). Among the findings of this work, it has to be highlighted the high number of antibiotic resistances displayed by some isolates of *S. epidermidis* (up to 10) and *S. hominis* (up to six) and the high percentage of MDR isolates, about 64% and 81%, respectively. Frequent resistance to several antimicrobials has been reported for these species in previous studies (Sahal and Bilkay, 2014; Deplano et al., 2016; Szczuka et al., 2016).

Streptococci constitute the second bacterial group involved in human infectious mastitis as suggested by the results of this study. Most streptococci isolated from mastitis samples belonged to the VGS, mainly to mitis and salivarius groups, as previously reported (Delgado et al., 2008; Arroyo et al., 2010; Mediano et al., 2017). These microorganisms are normally related to subacute and subclinical processes (WHO, 2000; Contreras and Rodríguez, 2011). Similarly to coagulase-negative staphylococci, VGS have been considered commensal microorganisms and, independent of their concentration and strain characteristics, they have been regarded as contaminants; as a consequence, their clinical relevance has often been questioned.

Accurate identification of VGS isolates at the species level is becoming an important issue to provide a better understanding of their potential pathogenicity and the increasing antibiotic resistance observed among them (Bruckner and Gigliotti, 2006; Doern and Burnham, 2010; Chun et al., 2015). The fact that many clinical and epidemiological data concerning these organisms usually refer to the entire VGS group rather than to a single species (Gordon et al., 2002) is an obstacle for knowing the role of each species of the group. Recently, the application of the MALDI-TOF MS has emerged as a useful, rapid and cost-effective method for the identification of these clinically relevant microorganisms (Kärpänoja et al., 2014; Angeletti et al., 2015; Martín et al., 2016).

Knowledge of antibiotic resistance in VGS is more limited than for staphylococci. Indeed, the antimicrobial susceptibility of streptococci isolates recovered from human mastitis has not been described to date. The increasing resistance of VGS against penicillin, erythromycin and tetracycline has become a concerning issue in the clinical practice (Gordon et al., 2002; Bruckner and Gigliotti, 2006; Ergin et al., 2006; Doern and Burnham, 2010; Chun et al., 2015), which is according with the high levels of resistance to these antimicrobials found among VGS isolates from human mastitis cases. Erythromycin-resistant VGS isolates usually are also resistant to tetracycline (Ergin et al., 2006), since the resistance genes to these antibiotics are often found on the same mobile genetic element (Cerdá Zolezzi et al., 2004; Doern and Burnham, 2010).

There were significant differences regarding the antibiotic susceptibility profiles among the different VGS species isolated in this study. *S. parasanguinis* exhibited a significantly higher resistance to benzylpenicillin, ampicillin, and tetracycline than *S. salivarius* and *S. mitis/oralis*, with antibiotic resistance percentages consistent with those reported by Chun et al. (2015). In relation to *S. salivarius*, the resistance to penicillins and levofloxacin was also higher than that demonstrated by *S. mitis/oralis.* These results are in disagreement with previous studies that have addressed higher antibiotic resistance in *S. mitis/oralis* compared with other streptococci (Han et al., 2006). Results concerning MDR revealed than 51% of the *S. parasanguinis* isolates were MDR compared to 39% of *S. salivarius* and 35% of *S. mitis/oralis*. This is in contrast with previous studies describing that *S. mitis/oralis* was more resistant to antibiotics than other VGS species (Doern and Burnham, 2010). *S. parasanguinis* and *S. salivarius* may play a relevant role in antibiotic resistance transmission among VGS involved in human mastitis, a fact that deserves to be addressed in the future.

The resistance patterns reported for staphylococci and streptococci in this study are concordant with commonly prescribed antibiotics, which supports that selective pressure from the use of antibiotics is a main factor in the development of antibiotic resistance (Fair and Tor, 2014), as well as in the molecular changes that enhance the virulence and biofilm-forming ability of different microorganisms (Dancer, 2004; Sahal and Bilkay, 2014). Therefore, the development of antimicrobial resistance among staphylococci and VGS isolated from human mastitis has serious implications for the treatment of this condition. Benzylpenicillin and erythromycin resistance is so high among these bacterial groups that they and closely related molecules should not be considered as a treatment of choice. On the other hand, the prevalence of oxacillin resistance among *S. epidermidis* isolates must also be contemplated as an issue of clinical relevance in relation to treatment options. Regarding streptococci, the differences in antimicrobial resistance pattern among species must be taken into consideration to decide the most appropriate antimicrobial therapy.

In practice, *in vitro* sensitivity to a given antibiotic does not mean necessarily that such antibiotic will be efficient for the treatment of mastitis. It must be highlighted that, beside antimicrobial resistance, other factors may lead to failure of antibiotic therapy and persistence of infection and, among them, the biofilm formation ability of the strain involved is of utmost importance (Høiby et al., 2010; Büttner et al., 2015). *S. epidermidis* is commonly regarded as the most frequent causative agent of infections of indwelling medical devices (such as peripheral or central venous catheters), followed by *S. aureus*. Once the bacteria have entered the body, they use various virulence factors to facilitate interactions with host tissues and subvert the host’s immune system. Interestingly, lactating mammary glands contain an extraordinary complex net of ducts during lactation that provides an excellent physical support for biofilm formation and maturation. Biofilms are multicellular, surface-attached agglomerations of microorganisms and their regulation involves quorum-sensing systems and it is not yet completely understood. Previous epidemiological and genetic studies suggest that *S. epidermidis* isolates in the clinical environment differ from those obtained outside of medical facilities in terms of biofilm formation, antibiotic resistance, and the presence of mobile DNA elements (Otto, 2009). In this context, the comparison of several properties of 200 *S. epidermidis* strains isolated from women with mastitis with those displayed by 105 ones isolated from milk of healthy women revealed that the number of strains that contained biofilm-related genes and that showed resistance to oxacillin, erythromycin, clindamycin, and mupirocin was significantly higher among the strains isolated from mastitic milk (Delgado et al., 2009). The authors suggested that resistance to diverse antibiotics and a greater ability to form biofilms found among the clinical strains may explain the chronic and recurrent nature of this infectious condition.

The genera *Corynebacterium* and *Rothia* are increasingly recognized as emerging opportunistic pathogens (Bernard, 2012; Ramanan et al., 2014), but their relevance in human mastitis may be greatly underestimated. Nevertheless, corynebacteria are common mastitis agents in ruminants (Contreras and Rodríguez, 2011) and they are the main cause of human granulomatous mastitis (Taylor et al., 2003; Mathelin et al., 2005). *Rothia* spp. have emerged as opportunistic pathogens associated with serious infections including sepsis, endocarditis, and meningitis in immune-compromised patients (Ramanan et al., 2014; Abidi et al., 2016). *R. mucilaginosa*, the most common species of this genus isolated in this study, has been previously involved in prosthetic device infections, which could be related, as in the case of coagulase-negative staphylococci, to the organism’s ability to produce a biofilm (Bruminhent et al., 2013). In this study, *Corynebacterium* and *Rothia* were found in 11 and 22% of samples, respectively, which suggests an important role in human mastitis pathogenesis, according to a previous study (Mediano et al., 2017). In relation to antimicrobial susceptibility, the VITEK 2 technology used for AST in this study is not available for these bacterial genera yet, and therefore the analyses of their antimicrobial resistance profiles will be part of a future study. It is of utmost importance since many medically relevant species have shown broad-spectrum antibiotic resistance which has important therapeutic implications (Olender, 2013; Dobinson et al., 2015).

*Enteroccoccus* are not frequent etiological agents in human mastitis but they can be relevant in specific cases. Isolates of this genus were present in some samples of this study (3.7%), most of them belonging to *E. faecalis.* Treatment of enterococcal mastitis may be particularly challenging due to the wide variety of antibiotic mechanisms displayed by the genus (Sood et al., 2008; Kristich et al., 2014). The antimicrobial susceptibility patterns of enterococci isolated from human mastitis have not been reported to date. In this study, the high level resistance to aminoglycosides exhibited by about 20% of isolates, as well as the remarkable resistance to erythromycin (86%), is in concordance with previous studies (Kristich et al., 2014; Chakraborty et al., 2015; Osuka et al., 2016). On the contrary, all isolates showed high susceptibility to ampicillin as reported for enterococci isolated from mastitic bovine milk (Nam et al., 2010). It must also be addressed that about 7% of the *E. faecalis* isolates obtained in this study showed resistance to quinolones and vancomycin. Even though *E. faecalis* resistance is low against vancomycin, the emergence of vancomycin-resistant enterococci is a cause of concern, as it can be transferred to *S. aureus* (Sood et al., 2008).

## Conclusion

The findings of this work are relevant to identify trends in antimicrobial resistance among mastitis pathogens, including the emergence of MDR strains, and to implement a rational treatment for this disease based on microbiological analysis and antibiotic susceptibility profiling. Further work is in progress to analyze the value of implementing AST on the clinical outcomes of women treated for mastitis, as well as to investigate the genetic mechanisms underlying the antimicrobial resistance.

## Author Contributions

MM: database construction, data analysis and interpretation, and wrote the paper. RA: bacterial identification, antimicrobial susceptibility testing. IE-M: data analysis. LF, JR: designed the research and reviewed the paper.

## Conflict of Interest Statement

The authors declare that the research was conducted in the absence of any commercial or financial relationships that could be construed as a potential conflict of interest.

## Footnotes

1 http://www.r-project.org
