# Supplementary material for: Identification of Emerging Human Mastitis Pathogens by MALDI-TOF and Assessment of Their Antibiotic Resistance Patterns
Source: Front Microbiol. 2017 Jul 12;8:1258. doi: 10.3389/fmicb.2017.01258 (PMC5506187; doi:10.3389/fmicb.2017.01258)
Supplement: Supplementary file 1 [file Table_1.PDF]

Supplementary Material

Identification of Emerging Human Mastitis Pathogens by MALDI-TOF and assessment of their Antibiotic Resistance Patterns

Supplementary Table S1. Minimum inhibitory concentration (MIC) of 16 antimicrobial agents against *Staphylococcus aureus* isolated from milk samples from women suffering infectious mastitis (n = 140)

	**MIC (mg/L)**												
**Antibiotic**	**0.03**	**0.06**	**0.12**	**0.25**	**0.5**	**1**	**2**	**4**	**8**	**10**	**16**	**64**	**128**
Benzylpenicillin	12.1	3.6	2.1	2.1	**80**								
Oxacillin				80.7	16.4			**2.9**					
Gentamycin					100								
Tobramycin						98.6					**1.4**		
Levofloxacin			60	37.1	1.4				**1.4**				
Erythromycin				0.7	33.6	**51.4**			**14.3**				
Clindamycin				98.6					**1.4**				
Linezolid						15.7	83.6	0.7					
Daptomycin			15.7	60.7	15.7	7.1		0.7					
Teicoplanin					97.1	0.7	2.1						
Vancomycin						29.3	68.6	2.1					
Tigecycline			97.9	2.1									
Fosfomycin									96.4		0.7	**1.4**	**1.4**
Fusidic acid					98.6		1.4						
Mupirocin							100						
Rifampicin					99.3					0.7			

Boldface indicates isolates (%) categorized as resistant by *Clinical and Laboratory Standards Institute* criteria (CLSI, 2013)
