# Supplementary material for: Identification of Emerging Human Mastitis Pathogens by MALDI-TOF and Assessment of Their Antibiotic Resistance Patterns
Source: Front Microbiol. 2017 Jul 12;8:1258. doi: 10.3389/fmicb.2017.01258 (PMC5506187; doi:10.3389/fmicb.2017.01258)
Supplement: Supplementary file 2 [file Table_2.PDF]

Supplementary Material

Identification of Emerging Human Mastitis Pathogens by MALDI-TOF and assessment of their Antibiotic Resistance Patterns

Supplementary Table S2. Minimum inhibitory concentration (MIC) of 16 antimicrobial agents against *Staphylococcus epidermidis* isolated from milk samples from women suffering infectious mastitis (n = 435)

	**MIC (mg/L)**													
**Antibiotic**	**0.03**	**0.06**	**0.12**	**0.25**	**0.5**	**1**	**2**	**4**	**8**	**16**	**32**	**64**	**128**	**512**
Benzylpenicillin	7.4	**0.7**	**0.5**	**2.8**	**88.7**									
Oxacillin				54.6	**0.2**	**0.7**	**0.5**	**44**						
Gentamycin					85	0.9	0.2	8.3	**2.1**	**3.5**				
Tobramycin						68	20.9	3.7	**4.6**	**2.8**				
Levofloxacin			81.6	8	4.4	0.2		**3.4**	**2.3**					
Erythromycin				7.6	20.2	**4.6**	**0.2**		**67.4**					
Clindamycin				84.3	0.5		**0.7**	**1.2**	**13.4**					
Linezolid					1.4	84.4	13.8	0.2						
Daptomycin			0.9	18.1	73.7	6.1	1.2							
Teicoplanin					7.4	7.8	42.6	32	8.1	**2.1**				
Vancomycin					1.4	54.7	42.5	1.1	**0.2**					
Tigecycline			62.7	24.9	12.2	0.2								
Fosfomycin									91.5	1.1	0.7	**1.1**	**5.5**	
Fusidic acid					57.7	0.7	0.7	**5.1**	**12.4**	**8.5**	**14.9**			
Mupirocin							70.1	0.5				**1.4**		**28**
Rifampicin					99.5						**0.5**			

Boldface indicates isolates (%) categorized as resistant by *Clinical and Laboratory Standards Institute* criteria (CLSI, 2013)
