# Supplementary material for: Identification of Emerging Human Mastitis Pathogens by MALDI-TOF and Assessment of Their Antibiotic Resistance Patterns
Source: Front Microbiol. 2017 Jul 12;8:1258. doi: 10.3389/fmicb.2017.01258 (PMC5506187; doi:10.3389/fmicb.2017.01258)
Supplement: Supplementary file 3 [file Table_3.PDF]

Supplementary Material

Identification of Emerging Human Mastitis Pathogens by MALDI-TOF and assessment of their Antibiotic Resistance Patterns

Supplementary Table S3 Minimum inhibitory concentration (MIC) of 10 antimicrobial agents against *Streptococcus mitis/oralis* isolated from milk samples from women suffering infectious mastitis (n = 215)

	CMI (mg/L)								
**Antibiotic**	**0.06**	**0.12**	**0.25**	**0.5**	**1**	**2**	**4**	**8**	**16**
Benzylpenicillin	29.8	14.9	**22.8**	**7.9**	**7.4**	**3.7**	**4.7**	**8.8**	
Ampicillin			57.2	**5.6**	**4.2**	**6.5**	**11.2**	**5.1**	**10.2**
Cefotaxime		66.5	3.3	7.9	5.1	**3.7**	**4.7**	**8.8**	
Ceftriaxone		45.6	22.3	6.5	9.8	**4.7**	**5.1**	**6**	
Levofloxacin			7	42.8	35.3	11.2	**1.4**	**0.5**	**1.9**
Erythromycin		26.8	3.1	0.5	**3.6**	**30.9**	**18.6**	**16.5**	
Clindamycin			88.8	0.9	**10.3**				
Linezolid						99.5	**0.5**		
Vancomycin		10.3	10.3	73.2	6.1				
Tetracycline			14	52.6	0.5	1.9	**4.2**	**0.5**	**26.5**

Boldface indicates isolates (%) categorized as resistant by *Clinical and Laboratory Standards Institute* criteria (CLSI, 2013)
