# Supplementary material for: Identification of Emerging Human Mastitis Pathogens by MALDI-TOF and Assessment of Their Antibiotic Resistance Patterns
Source: Front Microbiol. 2017 Jul 12;8:1258. doi: 10.3389/fmicb.2017.01258 (PMC5506187; doi:10.3389/fmicb.2017.01258)
Supplement: Supplementary file 4 [file Table_4.PDF]

Supplementary Material

Identification of Emerging Human Mastitis Pathogens by MALDI-TOF and assessment of their Antibiotic Resistance Patterns

Supplementary Table S4 Minimum inhibitory concentration (MIC) of 10 antimicrobial agents against *Streptococcus parasanguinis* isolated from milk samples from women suffering infectious mastitis (n = 82)

	**CMI (mg/L)**								
**Antibiotic**	**0.06**	**0.12**	**0.25**	**0.5**	**1**	**2**	**4**	**8**	**16**
Benzylpenicillin	9.8	14.6	**39**	**3.7**	**11**	**4.9**	**6.1**	**11**	
Ampicillin			28	**1.2**	**9.8**	**24.4**	**11**	**8.5**	**17.1**
Cefotaxime		57.3	4.9	8.5	7.3	**8.5**	**6.1**	**7.3**	
Ceftriaxone		52.4	13.4	7.3	7.3	**9.8**	**4.9**	**4.9**	
Levofloxacin			1.2	14.6	45.1	24.4	**11**	**1.2**	**2.4**
Erythromycin		19.2				**47.4**	**14.1**	**19.2**	
Clindamycin			80.5	**6.1**	**13.4**				
Linezolid						100			
Vancomycin		5.1	12.7	75.9	6.3				
Tetracycline			3.7	20.7	9.8	14.6	**19.5**	**2.4**	**29.3**

Boldface indicates isolates (%) categorized as resistant by *Clinical and Laboratory Standards Institute* criteria (CLSI, 2013)
