# Supplementary material for: Identification of Emerging Human Mastitis Pathogens by MALDI-TOF and Assessment of Their Antibiotic Resistance Patterns
Source: Front Microbiol. 2017 Jul 12;8:1258. doi: 10.3389/fmicb.2017.01258 (PMC5506187; doi:10.3389/fmicb.2017.01258)
Supplement: Supplementary file 5 [file Table_5.PDF]

Supplementary Material

Identification of Emerging Human Mastitis Pathogens by MALDI-TOF and assessment of their Antibiotic Resistance Patterns

Supplementary Table S5. Minimum inhibitory concentration (MIC) of 10 antimicrobial agents against *Streptococcus salivarius* isolated from milk samples from women suffering infectious mastitis (n = 162)

	**CMI (mg/L)**								
**Antibiotic**	**0.06**	**0.12**	**0.25**	**0.5**	**1**	**2**	**4**	**8**	**16**
Benzylpenicillin	7.5	18.6	**37.3**	**8.1**	**14.9**	**6.8**	**1.9**	**5**	
Ampicillin			39.1	**16.1**	**5**	**10.6**	**21.7**	**3.1**	**4.3**
Cefotaxime		60.5	3.7	14.2	11.7	**4.3**	**1.9**	**3.7**	
Ceftriaxone		40.7	25.9	4.9	20.4	**3.7**	**3.1**	**1.2**	
Levofloxacin			1.9	2.5	34	40.1	**16.7**	**2.5**	**2.5**
Erythromycin		29.6	0.6		**0.6**	**37**	**9.3**	**22.8**	
Clindamycin			78.4	**4.9**	**16.7**				
Linezolid						100			
Vancomycin		0.6	0.6	20.4	75.8	**0.6**	**1.3**	**0.6**	
Tetracycline			16	59.9	1.2	1.9	**0.6**		**20.4**

Boldface indicates isolates (%) categorized as resistant by *Clinical and Laboratory Standards Institute* criteria (CLSI, 2013)
