# Supplementary material for: Identification of Emerging Human Mastitis Pathogens by MALDI-TOF and Assessment of Their Antibiotic Resistance Patterns
Source: Front Microbiol. 2017 Jul 12;8:1258. doi: 10.3389/fmicb.2017.01258 (PMC5506187; doi:10.3389/fmicb.2017.01258)
Supplement: Supplementary file 10 [file Image_5.PDF]

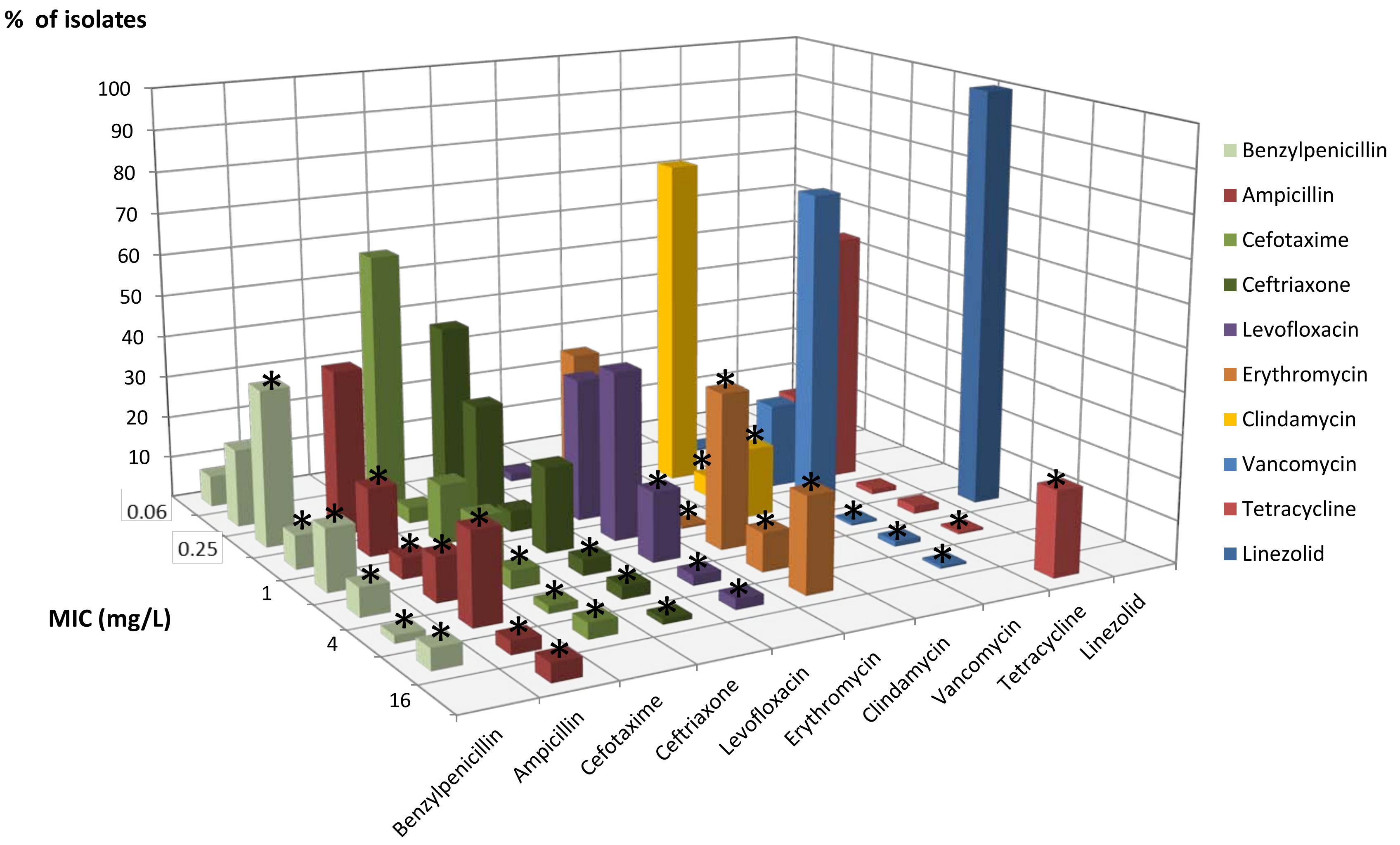

Supplementary Figure S5. Minimum inhibitory concentration (MIC) values of 10 antimicrobial agents against *Streptococcus salivarius* isolated from milk samples of women suffering from infectious mastitis (*n* = 162). Bars with asterisks represent isolates categorized as resistant by *Clinical and Laboratory Standards Institute* criteria (CLSI, 2013).
